# Genome-wide analysis of signal peptide functionality in *Lactobacillus plantarum *WCFS1

**DOI:** 10.1186/1471-2164-10-425

**Published:** 2009-09-10

**Authors:** Geir Mathiesen, Anita Sveen, May Bente Brurberg, Lasse Fredriksen, Lars Axelsson, Vincent GH Eijsink

**Affiliations:** 1 Norwegian University of Life Sciences, Center for Molecular Microbiology, Department of Chemistry Biotechnology and Food Science, Chr. M. Falsensvei 1. P.O. Box 5003, N-1432 Ås, Norway; 2 Norwegian Institute for Agricultural and Environmental Research, Plant Health and Plant Protection Division, Høgskoleveien 7, 1432 Ås, Norway; 3 Norfima Mat, Osloveien 1, N-1430 Ås, Norway

## Abstract

**Background:**

*Lactobacillus plantarum *is a normal, potentially probiotic, inhabitant of the human gastrointestinal (GI) tract. The bacterium has great potential as food-grade cell factory and for *in situ *delivery of biomolecules. Since protein secretion is important both for probiotic activity and in biotechnological applications, we have carried out a genome-wide experimental study of signal peptide (SP) functionality.

**Results:**

We have constructed a library of 76 Sec-type signal peptides from *L. plantarum *WCFS1 that were predicted to be cleaved by signal peptidase I. SP functionality was studied using staphylococcal nuclease (NucA) as a reporter protein. 82% of the SPs gave significant extracellular NucA activity. Levels of secreted NucA varied by a dramatic 1800-fold and this variation was shown not to be the result of different mRNA levels. For the best-performing SPs all produced NucA was detected in the culture supernatant, but the secretion efficiency decreased for the less well performing SPs. Sequence analyses of the SPs and their cognate proteins revealed four properties that correlated positively with SP performance for NucA: high hydrophobicity, the presence of a transmembrane helix predicted by TMHMM, the absence of an anchoring motif in the cognate protein, and the length of the H+C domain. Analysis of a subset of SPs with a lactobacillal amylase (AmyA) showed large variation in production levels and secretion efficiencies. Importantly, there was no correlation between SP performance with NucA and the performance with AmyA.

**Conclusion:**

This is the first comprehensive experimental study showing that predicted SPs in the *L. plantarum *genome actually are capable of driving protein secretion. The results reveal considerable variation between the SPs that is at least in part dependent on the protein that is secreted. Several SPs stand out as promising candidates for efficient secretion of heterologous proteins in *L. plantarum*. The results for NucA provide some hints as to the sequence-based prediction of SP functionality, but the general conclusion is that such prediction is difficult. The vector library generated in this study is based on exchangeable cassettes and provides a powerful tool for rapid experimental screening of SPs.

## Background

*Lactobacillus plantarum *is a Gram-positive lactic acid bacterium (LAB) with a long tradition in food fermentation, and is therefore Generally Regarded As Safe (GRAS status). This microbe is found in many ecological niches including naturally fermented food and decaying plant materials. Furthermore, *L. plantarum *is a normal inhabitant of the human gastrointestinal (GI) tract [1]. The complete genome sequence of *L. plantarum *WCFS1 has been determined [2], and tools for genetic engineering are available [3-7]. *L. plantarum *is adapted to survive in the harsh conditions of the GI-tract, as has been illustrated by recent genome-wide gene expression studies of the response of the bacterium to (mouse) GI-tract conditions [8,9]. Both the potential probiotic effects of *L. plantarum *and the high survival rate during the passage of the GI-tract make this bacterium a promising candidate as a vehicle for *in situ *delivery of therapeutically interesting proteins [10]. The general potential of LAB as *in situ *delivery vehicles for biomolecules is well recognized. For example, a recent phase I trial study has indicated that Crohn's disease patients benefit from treatment with a genetically modified *Lactococcus lactis *secreting human interleukin 10 [11]. Promising results have been obtained with LAB that secrete or anchor antigens to the cell (recently reviewed by Wells and Mercenier [12]; see also [13]).

Bacteria use several pathways for protein export to the membrane, the cell wall or the medium [14]. Many proteins follow the Sec-dependent pathway and are synthesized as precursors with an N-terminal signal peptide that directs the protein to the Sec translocation machinery. In the case of Sec-dependent secreted proteins, the signal peptide is cleaved off during or shortly after the translocation [15-17]. The genome of *L. plantarum *WCFS1 codes for more than 200 proteins that contain an N-terminal signal peptide. About 100 of these proteins contain a potential signal peptidase I cleavage site, and are thus likely to be secreted to the culture medium or anchored to the cell wall [2,18]. For the large majority of the proteins whose secretion is directed by these signal peptides experimental data showing functional properties are lacking. Using bioinformatics, some of the proteins were predicted to be enzymes or to be involved in adherence to host components [18].

The possibility to secrete heterologous proteins in *L. plantarum *or other LAB has been addressed in several studies [5,19-25]. So far, engineered secretion in *L. plantarum *has mostly been based on the use of heterologous signal peptides. The most widely exploited heterologous signal peptides are those from the *L. lactis *Usp45 protein [26-28], the *Streptococcus pyogenes *M6 protein [5,27,29], and the *L. brevis *S-layer protein [20,24], as well as signal peptides from different microbial amylases [4,30]. When aiming for the construction of genetically engineered *L. plantarum *strains for human consumption, there is a need for the use of homologous signal peptides since this limits the use of foreign DNA and since homologous signal peptides may lead to more efficient secretion. One key problem in selecting suitable signal peptides is the difficulty in predicting their efficiency on the basis of their sequence only (see below).

In this study we present the first genome-wide experimental analysis of the functionality of SPs from lactic acid bacteria. We have conducted a functional analysis of 76 of the 93 signal peptides from *L. plantarum *WCFS1 that were predicted by Kleerebezem et al. [2] to be processed by signal peptidase I. Seventeen of the 93 SPs were discarded from the study, primarily because the prediction of the cleavage site was ambiguous. To study the functionality of the signal peptides, they were used to direct secretion of a nuclease (NucA) from *Staphylococcus aureus *and, for a subset, an amylase (AmyA) from *Lactobacillus amylovorus*. This screening revealed large variation in signal peptide functionality and led to identification of some homologous signal peptides that yielded high secretion levels in *L. plantarum*. Although we generally found little correlation between signal peptide sequence properties and secretion results, our genome-wide data do suggest some criteria that may be used to increase the likelihood of selecting signal peptides (SPs) that yield efficient secretion of heterologous proteins.

## Results

### Library construction

Kleerebezem et al. [2] identified 93 proteins with putative signal peptidase I cleavage sites in the genome of *L. plantarum *WCFS1. In this study we ran all the 93 protein sequences through the web-based SignalP 3.0 program, using both the neural network (NN) and hidden Markov model (HMM) algorithms to predict putative cleavage sites [31]. The two algorithms yielded the same conclusions for 78 SPs and these were selected for further studies. Two of the 78 sequences were omitted from the SP library, one (Lp_0374) because it's coding DNA contains a *Sal*I site and one because of cloning problems (Lp_0946). An overview of the 76 remaining SPs and two additional heterologous SPs (M6 & Usp45) included in the library is presented in additional file 1.

The length of the selected SPs varies from 24 (several proteins) to 57 (Lp_2796) residues. The large majority of the SPs (61 of 76) had lengths between 24 and 36 residues, and only one sequence was predicted to be longer than 50 residues (Lp_2796). Analyses of bacterial SPs have shown predominance for alanine at positions -3, -1 and +1 relative to the cleavage site [17,32,33]. Seventeen of the 76 selected sequences have the consensus Ala-X-Ala↓Ala cleavage site, whereas 47, 74 and 33 of the SPs contain an Ala in the -3, -1 and +1 positions, respectively. At position -2, 15 different residues are present, both small non-polar, polar and charged. The most dominant residue at the -2 position is glutamine which is present in 17 of the sequences. Those SPs that do not have Ala in -3 have small, non-polar residues at this position. In the +1 position Ala is most often replaced by Asp (25 SPs). Weblogos [34] for the predicted cleavage sites of all 76 SPs and some subgroups of SPs are presented in additional file 2.

The SP library was constructed by fusing SPs translationally to the start codon of the *sppA *gene downstream of its native inducible P_sppA _promoter using an *Nde*I restriction site, as described in Methods. At the C-terminal end of the SPs, two amino acids downstream of the predicted cleavage site were retained from the original protein. Because the SPs were fused to the NucA reporter protein by a 6 nucleotide linker creating a unique *Sal*I restriction site, every construct had a valine followed by an aspartic acid residue in positions +3 and +4 relative to the cleavage site. The staphylococcal NucA was selected as a reporter protein because of its stability, small size, easily measurable extracellular activity and because it has previously been successfully used as a reporter protein for secretion in lactic acid bacteria [26,35].

### Secretion capacity of the SP library

**Figure 1:**
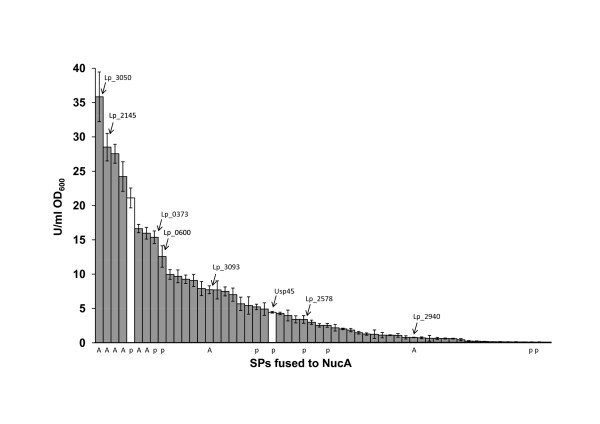
**Nuclease activities in supernatants of *L. plantarum *WCFS1 harbouring plasmids with different signal peptides (SPs)**. Signal peptides whose functionality in NucA secretion was tested in more detail by western blotting (see text) are marked with an arrow and are labeled with the corresponding gene code. The white bars represent the two heterologous SPs that were included in this study. Signal peptides that were also tested with the amylase reporter are marked with an A below the X-axis. Signal peptides whose functionality for NucA and AmyA has been addressed in a previous study [19] are marked with a p below the X-axis. Enzyme activities are expressed in Units per ml divided by OD_600_. All results are the mean of three independent experiments; the error bars indicate the standard deviation. Only the 58 SPs that led to an extracellular nuclease activity equal to or higher than 0.1 U/ml OD_600 _are shown. Extracellular nuclease activities for the complete SP library are given in additional file 1.

To measure the secretion capacity of the SPs in the library, induced cells were harvested at OD_600 _~1.7 (late logarithmic phase) and nuclease activity was measured in cell free supernatants. Figure 1 shows that there is large variation in the secretion capacity among the SPs. For 14 of the SPs extracellular nuclease activity was not significantly higher than the activity found for the construct with no signal peptide (0.02 U/ml.OD_600_; p < 0.05 by t-test; data in additional file 1). The other constructs yielded a continuum of activity levels spanning from close to 0.02 U/ml OD_600 _to the almost 1800-fold higher value of 35.8 U/ml OD_600 _obtained for the best signal peptide, Lp_3050. We also compared the secretion capacity of the SPs in the library to the secretion capacity of commonly used heterologous SPs derived from the Usp45 (*L. lactis*) and the M6 (*S. pyogenes*) proteins. Both heterologous SPs yielded secretion of NucA and the SP from M6 was among the better performing SPs (Figure 1).

**Figure 2:**
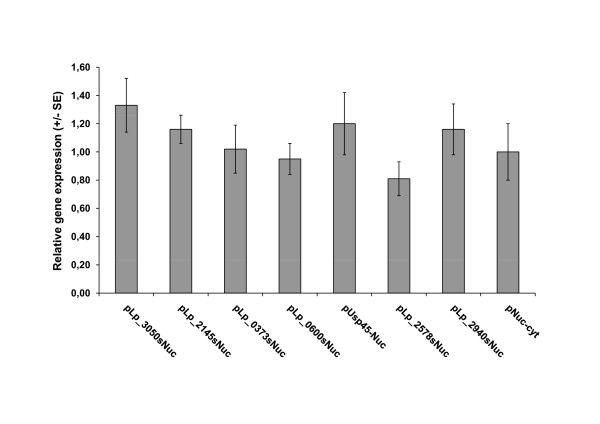
**Expression of NucA in recombinant *L. plantarum *strains as estimated using real-time PCR analyses**. The bars show expression ratios for *nucA*, calculated by comparing transcript levels in *L. plantarum *strains harbouring various constructs with the level in *L. plantarum*/pLp_3093sNuc, which was arbitrarily chosen as a control. The data are the mean of two independent experiments; standard errors (SE) are indicated. All Ct values were normalized against *gyrA *using the REST-program.

To check whether the variation in apparent secretion capacities was caused by differing transcription levels, we determined transcript levels in cultures from a subset of the cloned constructs using real-time PCR analyses. The results, depicted in Figure 2, show that there were no significant differences in transcript levels.

### NucA secretion efficiency of selected SPs in *L. plantarum*

**Figure 3:**
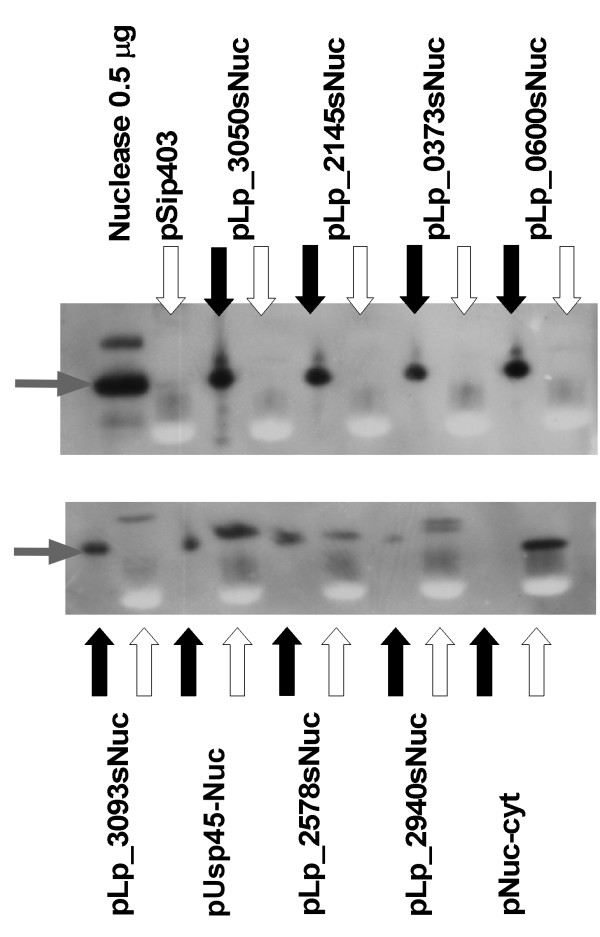
**Western blots for analysis of secretion efficiency**. White and black arrows indicate the cell lysate and supernatants fractions, respectively. Grey arrows indicate mature NucA. In addition to the results for eight constructs with signal peptides, the gels shows results for a construct driving cytoplasmic production of NucA (pNuc-cyt), a construct without the *nucA *gene (pSIP403, a construct for intracellular expression of *gusA*; [3]; only the cell lysate is shown for this construct) and a sample of pure mature NucA. For all the culture-derived samples, the sample size corresponded to 16 μl of the original culture harvested at an OD_600 _of approximately 1.7.

To investigate secretion efficiencies (i.e. the fraction of produced protein that is secreted), comparable amounts of cell and supernatant fractions were analyzed by Western blot experiments using a polyclonal antiserum against NucA. We selected eight SP constructs from the library covering a wide range of secretion capacities (Figure 1). Processed NucA could be detected in the supernatant of all selected clones (Figure 3), but not in the supernatant fraction of the control *L. plantarum *harboring the pNuc-cyt construct lacking a signal peptide. Both the fact that processing occurred and the lack of extracellular NucA in the pNuc-cyt control show that extracellular NucA detected in the transformants is not due to cell lysis. The amount of NucA detected in the supernatants by Western blotting generally corresponded well with the measured extracellular NucA activities shown in Figure 1.

Figure 3 shows that the secretion efficiency was close to 100% for the four SPs that yielded the highest extracellular activities, while secretion efficiencies were lower for the rest of the constructs. In these latter cases unprocessed NucA accumulated intracellularly. Using a sample of pure NucA as a standard, the amount of secreted NucA obtained with the pLp_3050sNuc plasmid was estimated to be in the range of 5 - 10 mg/l culture.

### Secretion of *L. amylovorus *α-amylase (AmyA)

In order to test the SPs' general usefulness to direct secretion, the six SPs leading to highest NucA secretion were also used to direct secretion of the 49 kDa N-terminal fragment of the α-amylase (AmyA) from *L. amylovorus *NRRL B-4549 [36,37]. For comparison, two additional SPs that led to lower extracellular NucA levels (Lp_3093 & Lp_2940) were included. The performance of the new constructs was analyzed by activity measurements (Table 1) and SDS-PAGE (additional file 3). All SP-containing constructs led to extracellular amylase activity but secretion efficiencies were well below 100% in all cases. The latter contrasts with the observations made for NucA, where secretion efficiencies for the better constructs approached 100% (Figure 3). The total AmyA activity (intra-plus extracellular) varied greatly among the constructs and generally the constructs yielding highest total activity displayed the lowest secretion efficiency. In line with previous observations [19], Table 1 shows that there generally is little correlation between the performance of an SP in NucA secretion and its performance in AmyA secretion. The trend in the data in Table 1 seems to be that SPs leading to intermediate secretion levels of NucA are among the best performers for AmyA, both with respect to the total level of extracellular activity and secretion efficiency.

**Table 1:** Secretion efficiency and α-amylase activity in recombinant *L. plantarum *WCFS1 harbouring various constructs^a^.

**Plasmids**	**Cell fractions** **(10^2 ^mU ml^-1 ^OD_600 _^-1^)**	**Culture supernatants** **(10^2 ^mU ml^-1 ^OD_600 _^-1^)**	**Secretion efficiency** **(%)^c^**	**Ranking number after NucA activity**
pLp_3050sAmy	7.2 ± 0.6	0.57 ± 0.04	7	1
pLp_2145sAmy	28.0 ± 3.6	0.92 ± 0.04	3	2
pLp_3189sAmy	8.7 ± 1.0	0.92 ± 0.03	10	3
pLp_3077sAmy	29.9 ± 5.1	0.43 ± 0.04	1	4
pM6sAmy^b^	53 ± 10	0.40 ± 0.17	0.7	5
pLp_0297sAmy	7.2 ± 2.6	3.4 ± 0.34	32	6
pLp_3117sAmy	6.8 ± 0.8	0.45 ± 0.1	6	7
pLp_0373sAmy^b^	5.3 ± 0.1	2.3 ± 0.1	30	8
pLp_0600sAmy^b^	1.8 ± 0.4	1.7 ± 0.2	49	9
pLp_3093sAmy	40.7 ± 2.0	0.30 ± 0.04	1	15
pLp_2958sAmy^b^	2.8 ± 0.8	1.5 ± 0.1	35	21
pUsp45sAmy^b^	29 ± 6	0.37 ± 0.14	1	23
pLp_2578sAmy^b^	2.4 ± 0.5	0.57 ± 0.2	19	27
pLp_2588sAmy^b^	2.0 ± 0.2	2.1 ± 0.1	51	30
pLp_2940sAmy	23.8 ± 6.4	(3.2 ± 0.3)^d^	n.d.^d^	41
pLp_3127sAmy^b^	28 ± 2	0.05 ± 0.01	0.2	56
pLp_1447sAmy^b^	1.5 ± 0.2	0.39 ± 0.08	21	57
pAmy-cyt	12.6 ± 4.6	0.05 ± 0.04	0	

^a ^Plasmids encoding SPs are ranked according to their capacity to secrete NucA, as shown in Figure 1 (best SP first). All results are the mean value of three biological replicates.

^b ^Data from Mathiesen et al [19]. Note that the selection of SPs in this previous study was more or less random and not based on the genome-wide overview of SP functionality that is presented here.

^c ^Based on the assumption that all intracellular, non-processed AmyA is equally active as secreted AmyA.

^d ^Apparent secretion is partly due to cell lysis, meaning that the secretion efficiency could not be calculated. See text for details.

**Figure 4:**
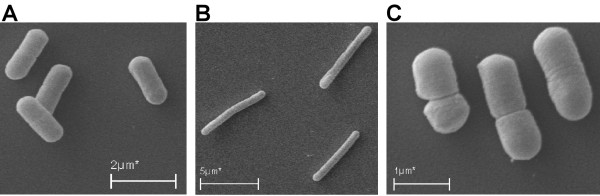
**Scanning electron microscopy images of recombinant *L. plantarum *WCFS1, harvested at ~OD_600 _1.7**. A, non-induced cells harboring pLp_2940sAmy; B, induced cells harboring pLp_2940sAmy; C, induced cells harboring pLp_0297sAmy.

Using silver stained SDS-PAGE gels (additional file 3), the secreted amylase could be detected in non-concentrated culture media and the relative intensities of the bands correlated well with the relative levels of enzyme activities. In the case of the pLp_2940sAmy construct, which leads to high total amylase levels, cell lysis was observed. Scanning electron microscopy showed that induced pLp_2940sAmy containing cells had an elongated shape that differed drastically from the shape of non-induced pLp_2940sAmy containing cells and induced cells containing other secretion constructs such as pLp_0297sAmy (Figure 4).

### Correlations between SP properties and secretion capacity for NucA

To search for a connection between SP sequence properties and secretion capacity, we carried out a series of calculations based on the SP sequences and compared the results with the NucA activity data. Tables 2 and 3 present the results of two types of group-wise analyses. In one analysis, the ten best performing SPs (1-10) were compared with SPs without significant extracellular activity (65-78). In the other analysis, the best performing half of the SPs (1-39) was compared with the least performing half (40-78). For each group mean values for the different properties were calculated and these values were then compared. For some of these analyses the SPs were divided into domains [17]. The N-domain was defined to span from the N-terminal methionine to the last positively charged residue in the N-terminal part of the SP, and had an average length of 8.3 residues. The rest of the SP is referred to as H+C domain, consisting of the hydrophobic H-region following the N-domain, followed by the C-domain that ends at the predicted cleavage site for the signal peptidase.

**Table 2:** Correlations between SP properties and measured extracellular NucA activities.^a^

**Ranked after NucA activity^b^**	**Lenght of SPs**	**pI of SPs**	**Hydro-phobicity^c^**	**Length of N-domain^d^**	**Net charge of the N-domain^d^**	**Charge/length of the N-domain^d^**	**Length of H-plus C-domains^d^**
1-10	34.5	9.8	2.98	7.2	3.1	0.5	27
65-78	32.8	10.1	2.31	9.9	3.0	0.4	23
1-39	31.6	10.3	2.77	7.4	2.9	0.5	24
40-78	31.1	10.1	2.26	9.1	3.0	0.4	22
1-78	31.3	10.2	2.52	8.3	3.0	0.4	23

^a^Average values for different properties were calculated for SPs grouped by NucA activity.

^b^Subgroups of the 78 SPs were made as indicated, with SPs being numbered by their ability to produce extracellular NucA activity, with SP number 1 having the highest activity; see text for further details.

^c ^The hydrophobicity of the SPs was estimated using the ProtScale program [66] using the Kyte & Doolittle scale [67] on the ExPASy Server . The numbers indicate the maximum hydrophobicity value, using a sliding window size of seven.

^d ^See text for definition of the domains. Nb. We verified that the length of the H+C domain is not correlated to the maximum hydrophobicity defined in footnote c (results not shown).

**Table 3:** Correlation between predicted transmembrane helices (TMH) in the SPs and measured extracellular NucA activities.

	**All SPs**	**SPs 1-39^a^**	**SPs 40-78^b^**
SPs with predicted TMH/Total number of SPs	62/78	38/39	24/39
SPs without predicted TMH (%)	21	2.6	38

^a^The best performing half of the SPs

^b^The least performing half of the SPs

Comparing the groups did not yield significant correlations between measured extracellular NucA activities and the following SP properties: isoelectric point of the complete SP, length of the SP, net charge or length of the N-domain, net charge/length of the N-domain, and the D-value provided by SignalP (see additional file 1 for raw data). However, the data showed that measured NucA activities were significantly (p < 0.05) correlated to SP properties as follows: (1) a positive correlation with SP hydrophobicity, found in both comparisons (1-10 vs 65-78 and 1-39 vs 40-78); (2) a positive correlation with the length of the H+C domain. A control analysis using only SPs that gave significant extracellular activity (1-32 versus 33-64) yielded the same correlations (results not shown).

Analysis of the sequence of the cleavage sites did not show any clear trends. In fact, the data did not suggest that the presence of the consensus sequence Ala-X-Ala↓Ala is particularly favourable. Both the A-X-A motif in front of the cleavage site and the A at position +1 were more abundant in the least performing half of the SPs. Only six of the 39 best performing SPs had the A-X-A↓A consensus sequence.

Previous studies have shown that SPs adopt α-helical conformations in interfacial environments such as cell membranes [38,39]. All 76 SPs as well as M6 and Usp45 were run through a web-based transmembrane helical prediction program, TMHMM Server v. 2.0 [40]. The prediction showed that 62 of the 78 SPs were predicted to adopt a transmembrane helix (TMH) structure. Interestingly, 97% of the 39 best performing SPs were predicted to contain a TMH, while this was the case for only 62% of the 39 worst performing SPs (Table 3; raw data see additional file 1). The observed secretion capacities showed no correlation with the length of the predicted TMH nor with the position of the predicted helix start (see additional file 1 for raw data). TMHMM also predicts the presence of SPs. Nine of the 78 SPs were not recognized as SPs by TMHMM and eight of these were all in the least performing half of the 78 tested SPs (additional file 1).

Interestingly, there also seems to be a weak correlation between the ability of an SP to drive secretion of NucA and the presence of motifs that keep the original cognate protein attached to the cell surface (LysM, LPxTG and C-terminal membrane anchoring motifs as predicted by Boekhorst et al. [18]; see additional file 1). Of the 41 proteins in the library harbouring one of these motifs, most have SPs that led to low secretion capacity for NucA (Table 4). 64% of the proteins belonging to the 39 worst performing SPs were predicted to contain a cell-wall anchoring motif, while this was the case for only 41% of the 39 proteins with the best performing SPs (Table 4 and additional file 1).

**Table 4:** Correlation between the presence of anchoring motifs^a ^(AM) in the natural cognate protein and measured extracellular NucA activities.

	**All SPs**	**SPs 1-39^b^**	**SPs 40-78^c^**
SPs with AM/Total number of SPs	41/78	16/39	25/39
SPs without AM (%)	47	59	36

^a^Investigated anchoring motifs: LPxTG, LysM domain and C-terminal

transmembrane domain, as annotated by the Secretome

database of *L. plantarum *[18].

^b^The best performing half of the SPs

^c^The least performing half of the SPs

## Discussion

We present a comprehensive study of putative SPs in the genome of *L. plantarum *WCFS1 for which SignalP predicted a unique cleavage site for signal peptidase I. The results provide genome-wide insight into SP functionality, new tools (vectors) for secretion of proteins using homologous SPs, and increased insight into the predictability of SP functionality on the basis of sequence only.

82% (p < 0.05) of the 76 tested SPs led to secretion of NucA. While this result may be taken to confirm that the 62 *L. plantarum *proteins containing these SPs indeed are secreted, it does not imply that the remaining 14 SPs do not function at all and that their cognate proteins are not secreted. SP functionality depends on which protein is being secreted [41,42], meaning that SPs that do not work for NucA may function when coupled to another protein (and, in principle, *vice versa*). Furthermore, in some cases prediction of the signal peptidase cleavage site may have been wrong, despite the unanimous prediction by the two Signal P algorithms (see also below). Indeed, comparison of the sequences of some of the seemingly non-functional SPs (see additional file 1) with what is known about cleavage site sequences (illustrated by the sequence logos in additional file 2) show that alternative cleavage sites are possible in some of these SPs. The detected levels of extracellular NucA varied by three orders of magnitude. Since the only difference between the constructs is the SP, the large differences in secretion capacity are due to variation in the SP, directly or indirectly. To try to unravel the causes of these variations we set up additional experiments and looked closer into the properties of the SPs.

Real-time PCR studies of cultures containing different constructs did not reveal significant differences in mRNA levels. This indicates that the large variation in secretion capacity observed for these constructs is not due to differences in transcription levels. This is an expected result, since the constructs contain identical transcription initiation and termination signals. Thus, the variation in secretion capacities must be governed by (inter-related) post-transcriptional factors such as secondary structure of mRNA, codon usage and translation efficiency, the interaction between the precursor protein and the translocation machinery, the efficiency of the signal peptidase for the SP in question, the rate of (non-desirable) intracellular and (desirable) extracellular folding, and possible interactions between the secreted protein and the bacterial cell wall [41,43-45].

Although the Western blot of Figure 3 provides only limited quantitative insight, the data do suggest that all *L. plantarum *transformants produced approximately equal amounts of NucA, meaning that all transformants experienced approximately equal "protein loads". The data show a (rough) correlation between translocation efficiency and the levels of secreted protein (Figure 3). One possible cause of variation in secretion efficiency is variation in the efficiency of SP processing. However, in their genome-wide study of *B. subtilis *SPs Brockmeier et al. [42] showed that the rate of precursor processing had limited effects on levels of extracellular reporter protein. Assuming a similar situation in *L. plantarum*, differences in the efficiency of the translocation process itself remain as the main cause of the variation in extracellular NucA levels.

Mutagenesis studies have confirmed that secretion levels in Gram-positive bacteria are not only affected by variation in the SPs [46-48] but also by variation in the N-terminal part of the mature protein [21,35]. Le Loir et al. [21] showed that negative charge in the N-terminal part of the secreted protein was beneficial for secretion. The NucA variants in the present study varied only with respect to residues +1 and +2 and we did not observe correlations between the character of these residues and secretion performance of the SP. The very efficient Lp_3050 sequence has a basic residue (Lys) at position +2 which is unexpected on the basis of the conclusions drawn by Le Loir et al. [21]. Taking into account the above considerations, it is likely that the variation in the secretion of NucA observed in this study is caused by the variation of the SP only and its effect on the interaction between the precursor and the translocation machinery.

The translocation process is a complex process which involves many interactions that are affected by the characteristics of both the SP and the protein. It is conceivable, that SPs are evolutionary adapted to their cognate protein to ensure efficient and controlled secretion. The importance of the protein part is clearly shown in both the present study and a previous genome-wide study on SPs from *B. subtilis *[42], which show that the efficiency of many SPs depends on the reporter protein. Thus, high secretion efficiency requires an optimal combination between the SP and the target protein. Recent studies suggest that SP function may be much more complex than previously thought, and may direct surface proteins to different subcellular locations [49-51]. Clearly, such underlying complexities in SP functionality, will weaken correlations between SP sequence properties and secretion levels.

Several studies have shown that changes in hydrophobicity of the H-domain can affect the secretion capacity [47,52,53] and this is indeed one of the correlations that we discovered in the present genome-wide study. However, in a study of 148 SPs from *B. subtilis *[42] no such correlation was found. In the present study, we also identified a clear correlation between a predicted transmembrane helix by the programme TMHMM and high secretion capacity. On the basis of our results, running TMHMM seems one of the best ways to select SPs that are likely to perform well, and this analysis should thus be performed next to SignalP. In addition, the length of the H+C domain should also be taken into account when selecting an SP. It is interesting to note that SPs from proteins that are thought to be anchored to the cell wall tend to perform less well than other SPs. It is conceivable that these proteins do not require high translocation efficiencies, since they are not meant to be actively secreted to the surrounding media and therefore may be produced at lower levels than released proteins.

In this study, we have based the prediction of signal peptides on the original analysis of the *L. plantarum *genome as described by Kleerebezem et al [2] and we have used SignalP 3.0 to check and predict the cleavage sites. Clearly, the annotation of the *L. plantarum *genome will evolve as bioinformatic tools evolve and today's annotation, e.g. with respect to the subcellular localization of proteins, will differ from the one published in 2003. The most accurate prediction of extracellular protein localization in *L. plantarum *WCFS1 is found in the Secretome database [18]. Another prediction tool is the newly developed Locate P [54] that combines existing predictors and produces genome-wide predictions for the subcellular locations of bacterial proteins in a fully automated manner. Predictions based on both methods/databases for the 78 proteins relevant for this study are included in additional file 1 and show several differences. For example, all but one (Lp_1524) of the selected SPs are predicted to be cleaved by SPaseI in the Secretome database, whereas Locate P predicts such cleavage only for 63 of the SPs. The present set with experimental data may be used to evaluate prediction quality and, hopefully, to improve prediction methods. Our data show that the SPs of several proteins predicted to be N-terminally anchored by LocateP lead to efficient secretion of NucA, meaning that they are cleaved by SPaseI as predicted by SignalP and according to the prediction in the Secretome database. Likewise, several proteins predicted to be multi-membrane proteins according to Locate P contain SPs that are quite efficient for NucA secretion.

To test the general performance of the SPs we replaced NucA with AmyA in selected constructs. When produced at levels applied in this study, AmyA seems to be difficult to handle for *L. plantarum*. Secretion efficiencies were below, often far below, 100% for all constructs. Table 1 shows that the AmyA constructs lead to highly variable overall production levels, creating a complicating variable that was less prominent in the studies with NucA. Previous studies have shown that overexpressed amylase can be difficult to handle for *B. subtilis *and induce stress reactions [55,56]. Table 1 also shows that high production levels of AmyA correlate with low secretion efficiencies, suggesting that the translocation machinery is overloaded. In addition to slow or blocked translocation, secretion stress may cause intracellular or extracellular proteolytic degradation [41,56]. Proteolytic degradation was not analyzed because of the lack of a suitable antibody for AmyA. The stress caused by AmyA expression is illustrated by cells harbouring the pLp_2940sAmy construct that leads to high levels of AmyA production. These cells showed impaired growth (data not shown), cell lysis and a change in morphology (Figure 4). Lp_2940 did not perform very well for NucA (rank 41) and it does not have the properties that are typical for SPs that work well with NucA (see additional file 1). It is possible that the combination of a high production level with an unfavourable SP stressed the cells to the extent that lysis occurred. All in all, our observations with AmyA indicate that this protein is not a suitable reporter to search for characteristics in SP-sequences that correlate to secretion capacity.

## Conclusion

The present study shows that at least 82% of the tested putative signal peptidase I-dependent SPs in the genome of *L. plantarum *WCFS1 indeed functions as a signal for secretion. The results reveal considerable variation in SP performance that is at least in part dependent on the protein that is secreted. We identified correlations between SP sequence and SP performance which may be used for pre-selecting promising SPs, but the general conclusion is that prediction of SP performance is difficult. The lack of predictability suggests that sequence differences between SPs at least in part relate to other (potential) aspects of SP functionality, such as spatial and temporal regulation of protein production and secretion. As it stands, secreting a protein of interest at the highest possible levels in *L. plantarum *will require experimental screening of SPs. The library constructed in this study provides an easy to use tool for rapid experimental screening since it is based on exchangeable cassettes.

## Methods

### Bacterial strains and growth conditions

*Escherichia coli *TOP10 (Invitrogen, Carlsbad, CA, USA) cells were grown in BHI broth (Oxoid Ltd., Hampshire, England) at 37°C with shaking. *L. plantarum *WCFS1 [2] was grown in MRS broth (Oxoid) at 30°C without agitation. Solid media were prepared by addition of 1.5% (w/v) agar. Antibiotics were added as follows: for *E. coli*, kanamycin 100 μg/ml and erythromycin, 200 μg/ml; for *L. plantarum*, erythromycin 5 μg/ml.

### Standard genetic techniques and transformation

Primers used in this study were purchased from Operon Biotechnologies GmbH (Cologne, Germany) and are listed in additional file 4. Chromosomal DNA from *L. plantarum *was isolated using the E.N.Z.A Bacterial DNA kit (Omega Bio-Tek. Inc. Doraville, GA) by following the protocol provided by the manufacturer. Mutanolysin, 15 U/ml, was added to the cell lysis step in this protocol. All signal sequences were amplified from chromosomal DNA using Phusion polymerase (New England Biolabs, Inc., Ipswich, MA). The PCR fragments were isolated from a 3.5% NuSieve GTG agarose gel (Cambrex Bio Science Rockland, Inc. Maine) using the NucleoSpin Extract II kit (Macherey-Nagel GmbH & Co, Düren, Germany) and subsequently sub-cloned into PCR-Blunt II TOPO vector (Invitrogen, Carlsbad, CA) following the protocol from the manufacturer. The sequences of all PCR-generated inserts were confirmed by DNA sequencing.

Chemically competent *E. coli *TOP10 cells were transformed by following the protocol of the manufacturer and lactobacilli were transformed according to Aukrust et al. [57].

### Cloning strategy

The gene expression system used in this study is based on the modular pSIP-vectors that contain a peptide-pheromone inducible expression system for use in *Lactobacillus *[3,58]. This system has recently been modified to allow secretion of proteins by adding a "signal peptide cassette" [19]. In this system the N-terminal end of the desired SP is translationally fused to the inducible Sakacin P promoter (P_*sppA*_) in a modified version of plasmid pSIP401, using a *Nde*I restriction site at the start codon. The C-terminal end of the SP followed by an additional two amino acids downstream of the predicted cleavage site is fused in-frame to the reporter protein via a Val-Asp linker that yields a unique *Sal*I site at the DNA level. This SP-cassette module permits easy exchange of the SPs by using *Nde*I-*Sal*I restriction cloning.

SP sequences were amplified using PCR with primer pairs (named after the gene number in the *L*. *plantarum *genome, see additional file 4) harbouring *Nde*I or *Sal*I sites and the resulting PCR fragments were cloned into PCR-Blunt II TOPO vector (Invitrogen). The SP-containing fragment was excised from the resulting plasmid by *Nde*I-*Sal*I restriction digesting and ligated into the 6.1 kb *Nde*I-*Sal*I fragment of pUsp45-Nuc[19], yielding constructs for secretion of NucA. Some selected SPs were also ligated into the 6.9 kb *Nde*I-*Sal*I fragment of pUsp45-Amy [19], yielding constructs for secretion of AmyA. All SPs used for making constructs are listed in additional file 1. As controls we used plasmids pNuc-cyt and pAmy-cyt [19] which direct production of non-secreted NucA and AmyA, respectively.

### Nuclease and amylase assays

Freshly inoculated cultures of *L. plantarum *WCFS1 harbouring a pSIP-derived plasmid (MRS, 30°C, 5 μg/ml erythromycin) were induced at an OD_600 _of 0.3 by adding the inducing peptide for sakacin P production [59] to a final concentration of 25 ng/ml. Cells were harvested in late-logarithmic phase at an OD_600 _of approximately 1.7. NucA activity in the supernatants was measured using the procedure described by Heins et al. [60]. The assay is based on release of acid soluble oligonucleotides from Calf Thymus DNA (Worthington, Lakewood, NJ, USA). One unit of nuclease activity corresponds to an activity generating an ΔOD_260 _of 1 per min under the conditions of the assay.

Amylase activity in the supernatant was measured directly using the Phadebas kit (Magle Life Sciences, Lund, Sweden) according to the manufacturer's procedure, with the following modifications: the sample volume was 0.5 or 0.05 ml and the reactions were conducted at 50°C. To measure intracellular amylase activity, the cells were harvested, washed once with dH_2_O, and resuspended in one-fifteenth of the original volume. The cells were disrupted by glass beads (Sigma) using FastPrep-24 instrument (MP Biomedicals, Solon, OH) to obtain crude protein extracts. Amylase activities were calculated using a standard curve made with α-amylase purchased from Sigma (product number A-6380), using the Unit definition provided by Sigma.

### SDS-PAGE

Proteins in cell-free supernatants and intracellular proteins were visualized by running 10% NuPAGE Novex Bis-Tris gels using MOPS as running buffer (both Invitrogen). Proteins were visualized using the SilverSNAP Stain for Mass Spectrometry kit from Pierce (Rockford, IL, USA) for extracellular proteins and Coomassie Brilliant Blue for intracellular proteins.

### Western blot analysis

For Western blotting 2 ml cell cultures were handled essentially as described by Piard et al.[61]. The proteins from the supernatant were precipitated by adding 400 μl ice-cold 80% (v/v) trichloroacetic acid (TCA) to 1.6 ml supernatant. The solution was incubated on ice for 30 min and the resulting precipitate was collected by centrifugation at 4°C for 10 min at 16 000 × *g*. The precipitate was washed with 300 μl ice-cold acetone and recentrifuged. After freeze drying, the protein pellet was dissolved in 25 μl NuPAGE LDS sample Buffer, 10 μl NuPAGE Reducing Agent (both Invitrogen) and 65 μl 10 mM Tris-HCl buffer (pH 8).

To extract intracellular protein, the cell pellets were washed once with TES-buffer (25% w/v sucrose, 1 mM EDTA and 50 mM Tris-HCl, pH 5.8). The cell wall was then partially digested by adding 500 μl TES buffer containing lysozyme (16 mg/ml), mutanolysin (60 U/ml) and RNase (0.5 mg/ml) (all from Sigma-Aldrich Inc, St. Louis, MO). After incubating the cell suspension for 1 hour at 37°C, protoplasts were collected by centrifugation at 15 000 × g for 3 min. The protoplasts were then lysed with 85.5 μl TES buffer containing, 12,5 μl 10% (w/v) sodium dodecyl sulphate (SDS) and the solution volume was adjusted to 125 μl with 20 μl NuPAGE Loading buffer (Invitrogen) and 6 μl NuPAGE Reducing agent (Invitrogen). Samples were denatured at 100°C for 10 minutes.

One microliter samples were run on 10% NuPAGE Novex Bis Tris Gels (Invitrogen) using MES (Invitrogen) as running buffer. Electroblotting was performed by using the iBlot Dry Blotting System (Invitrogen) according to manufacturer's recommendations, with the exception of the nitrocellulose membrane being replaced by a PVDF membrane (BioRad Laboratories, Inc, Hercules, CA). Rabbit polyclonal anti-NucA antiserum against the peptide EFDKGQRTDKYGRG [62] was obtained from ProSci Inc. (Poway, CA) and used as recommended by the manufacturer. Immunodetection was performed using a horseradish peroxidase-conjugated (HRP) goat anti-rabbit antibody (Bio-Rad) and the enhanced chemiluminescent kit from Pierce (Rockford, Il).

### Quantitative real-time PCR

Total RNA was isolated from cell cultures harvested at OD_600 _~1.7 using the RNeasy Mini Kit (QIAGEN) with on-column digestion of DNA with RNase-Free DNase Set (QIAGEN). After harvesting, cell pellets (from 0.5 or 1 ml of culture) were resuspended in 350 μl RLT buffer (RNeasy Mini Kit) containing 0.1% (v/v) β-mercaptoethanol (Sigma). Cells were directly transferred to FastPrep tubes (MP Biomedicals) containing glass beads (≤106 micron, Sigma) and 300 μl chloroform, and subsequently disrupted using a FastPrep-24 instrument (MP Biomedicals). After a short centrifugation, the water-phases from each sample were transferred to a new RNase free tube and centrifuged at 16 000 × *g *for 2 min. The supernatant was mixed with 250 μl ethanol and subsequently added to an RNeasy spin column. Further steps were performed according to the procedure of the RNeasy Mini Kit (QIAGEN). After RNA isolation, an additional DNase treatment was performed using TURBO DNase (Applied Biosystems, Foster City, CA) following the manufacturer's instructions. RNA concentrations were quantified using the NanoDrop spectrophotometer (Thermo Fisher Scientific Inc, Waltham, MA) and the quality of the RNA was assessed using the RNA 600 Nano LabChip kit and the Bioanalyzer 2100 (Agilent Technologies, Inc, Santa Clara CA). Control of residual chromosomal DNA from the total RNA isolation was performed on DNase treated samples. RNA was isolated from two independent cultures of each transformant, and these were analyzed as independent replicates throughout the real-time PCR procedure.

Synthesis of cDNA was performed using the Superscript III kit (Invitrogen) according to the manufacturer's instructions. Five-hundred nanogram total RNA and 100 ng random primers (Invitrogen) were used in each reaction.

All real-time PCR amplifications were performed using a 7900 HT Fast Real-Time PCR system (Applied Biosystems) with standard block, and data were analyzed using the Sequence Detection Systems software (Applied Biosystems).

PCR efficiencies (E) for the primer pairs for *nucA *and the reference gene *gyrA *(see additional file 4) were calculated from the slope of standard curves consisting of the amplification results from five 10-fold dilutions of a pool of cDNA samples, where E = 10^-1/slope^[63]. The *gyrA *gene was chosen as reference gene because it is known to be constitutively and stably expressed under various conditions in lactic acid bacteria [64]. The PCR program consisted of an initial denaturation step at 95°C for 10 min., followed by 40 cycles of 95°C for 15 s and 60°C for 1 min. Amplification was followed by melting curve analysis and determination of melting temperature for the PCR products, as a control of amplification specificity. Each PCR reaction contained 400 nM of gene-specific primers and 2 μl diluted (25×) cDNA in a total volume of 25 μl SYBR Green PCR Master Mix (Applied Biosystems). All reactions were assessed in triplicate. Relative expression of *nucA *is based on the ratio of the *nucA *transcript versus the reference gene transcript (*gyrA*), in cultures with cells containing the specific *nucA *construct, and was calculated using the relative expression software tool (REST) [65]. A randomly selected *nucA *construct (pLp_3093sNuc) was used as the control for all other samples in REST calculations, and expression ratios were calculated accordingly. The expression ratio results were tested for significance by a pair wise fixed reallocation test using REST [65].

### Scanning electron microscopy

Cells were harvested at OD_600_~1.7 by centrifugation at 2000 × g for 3 min and subsequently washed with 2 ml 0.9% (w/v) NaCl. The suspensions were centrifuged and the resulting pellets were stored at -20°C until use. Immediately prior to the analyses, cells were thawed on ice for 20 min and suspended in 1 ml 0.1 M Tris-HCl (pH 7.5). For scanning electron microscopy, several drops of cell suspension were transferred to glass cover slips coated with poly-L-Lysine. The cover slips were washed twice in 0.1 M Tris-HCl (pH 7.5) to remove excess of cells. Dehydration was performed by immersing the slides in a series of ethanol solutions (70, 90, 96, and 4 times in 100% ethanol). The cover slips were placed in a critical point drier (CPD 030, Bal-Tec, Balzers, Lichtenstein), mounted on Al-stubs using double faced carbon tabs (Agar Scientific, Essex, England), and subsequently coated with approximately 500 Å Pt in a SC7640 sputter coater (Quorum Technologies Ltd, Newhaven, U.K.)). The dried bacteria were analyzed in a Zeiss EVO-50 (Zeiss, Jena, Germany) scanning electron instrument at 10 kV.

### Analysis of signal peptides

Signal peptide cleavage sites were predicted using the SignalP 3.0 server [31,32], which is accessible at . The Secretome database of *L. plantarum *WCFS1 was assessed at [18]. LocateP was assessed at . [54]. Transmembrane predictions of the signal sequences were performed using the TMHMM Server v. 2.0 [40], which is accessible at . The hydrophobicity of the SPs was estimated using the ProtScale program [66] and the Kyte & Doolittle scale [67] on the ExPASy Server , using a sliding window of seven residues. Composition maps were made using the WebLogo application [34] which is accessible at .

## Authors' contributions

GM, LA and VE developed the initial concept for this study. GM, LA and VE participated in experimental design and coordination of the study. AS and GM constructed the library, carried out the enzyme assays, western analysis and analyzed the signal peptides. GM and MBB carried out the real-time PCR analysis and LF carried out the scanning electron microscopy. GM made the WebLogos. GM and VE drafted the paper, implementing contributions from all other authors. All authors read, corrected and approved the final manuscript.

## Supplementary Material

Additional file 1 **List of signal peptides**. Properties of the signal peptides (SPs) used in the present study and NucA activity in the supernatants of cells harbouring SP-NucA constructs. Click here for file

Additional file 2 **WebLogos of signal peptides**. Frequency plots made with WebLogo [34], based on multiple alignment of the sequence starting13 residues upstream of the predicated cleavage site and ending two residues downstream. Click here for file

Additional file 3 **SDS-PAGE analysis of *L. plantarum *containing various constructs for amylase secretion**. Coomassie stained and Silver-stained SDS-PAGE gels of cell extracts and cell free supernatants of *L. plantarum *WCFS1 harbouring various amylase constructs. Click here for file

Additional file 4 **List of primers**. Primers used in real-time PCR experiments and for PCR-amplification of signal peptides from genomic DNA of *L. plantarum *WCFS1. Click here for file
