# Supplementary material for: Genome-wide analysis of signal peptide functionality in Lactobacillus plantarum WCFS1
Source: BMC Genomics. 2009 Sep 10;10:425. doi: 10.1186/1471-2164-10-425 (PMC2748100; doi:10.1186/1471-2164-10-425)
Supplement: Additional file 1 — List of signal peptides. Properties of the signal peptides (SPs) used in the present study and NucA activity in the supernatants of cells harbouring SP-NucA constructs. [file 1471-2164-10-425-S1.pdf]

Table S1. Properties of signal peptides used in the present study and NucA activty in the supernatant of cells harboring SP-NucA constr

No.	Signal peptide (SP)	Amino acids sequence of the SPs including 2 amino acids downstream of cleavage site	NucA activity U/ml OD_{600}	Lenght	pI	Length of N-domain[a]	Net charge of the N-domain[a]	Charge/Length of the N-domain	Length of H- pluss C-domains	Maximum hydrophobicity[b]	Predicted TMH in SP using TMHMM v. 2.0	Length of predicted helix (TMHMM)	Start of predicted helix (TMHMM)	Predicted to be a SP by using TMHMM v. 2.0 server	Cognate protein with LPXTG motif[c]	Cognate protein with LysM domain[c]	Cognate protein with C-terminal anchor[c]	Predicted cleavage site in LocateP[d]	Subcellular localization prediction in LocateP[d]	D-value wild type proteins	D-value NucA constructs	Signal peptide (SP)
1	Lp_3050	MKKFNFKTMLLLVLASCVFGVVVNVTTSLGPQTAITAQA-SK	35.84±3.62	39	9.8	7	3	0.4	32	3.01	1	23	9	1				QASKKLSQ	N-term	0.763	0.739	Lp_3050
2	Lp_2145	MKKINKLMILGMLVFGVTGATMINPEMTTAAHA-SA	28.51±2.01	33	9.7	6	3	0.5	27	2.92	1	18	7	1			1	None	Membrane	0.614	0.56	Lp_2145
3	Lp_3189	MRFAGKLKKVMIALVAAVTFSTGGLGIAGADLQAQA-AS	27.55±1.38	36	10.3	9	4	0.4	27	3.13	1	23	12	1				LQAQAAST	Secretory	0.748	0.694	Lp_3189
4	Lp_3077	MKKLMIAVTGLAMVATLGAAVPVTGITANA-AT	24.23±2.15	30	10.0	3	2	0.7	27	2.34	1	23	4	1				None	N-term	0.902	0.85	Lp_3077
5	M6	MAKNNTNRHYSLRKLKKGTASVAVALSVIGAGLVVNTNEVSA-RV	21.11±1.45	42	10.6	17	7	0.4	25	2.59	1	23	20	1	1			Not specified	LPxTG	0.552	0.57	M6
6	Lp_0297	MKEVRFWGLLLGLFVCLGAVIPLVSKA-DV	16.64±0.6	27	9.3	5	1	0.2	22	3.12	1	18	7	1				LVSKADVL	Secretory	0.639	0.596	Lp_0297
7	Lp_3117	MRRICKVLMVIISIILGSGAPLNMAIPPLLALA-AP	15.97±0.85	33	10.9	6	3	0.5	27	3.48	1	23	7	1				None	N-term	0.74	0.692	Lp_3117
8	Lp_0373	MYTENTGKHHRNGLPVWLLPLLVVISFWGVSQNIMVVDA-SS	15.38±0.92	39	6.7	11	3	0.3	28	2.86	1	23	13	1	1			None	Membrane	0.551	0.524	Lp_0373
9	Lp_0600	MMKHKNLLLLIIMSLGFCGMLLMFAPQISTLINPNASRVEA-DK	12.58±1.56	41	9.3	5	3	0.6	36	3.23	1	23	7	1				None	N-term	0.614	0.545	Lp_0600
10	Lp_3451	MKKIFWQLIAVVSIVMAGLTMTARA-DD	9.96±0.71	25	11.2	3	2	0.7	22	3.14	1	20	4	1	1			Not specified	LPxTG	0.774	0.651	Lp_3451
11	Lp_3115	MQKRLRLSLGMLLAVVASLLMMGQVASA-DQ	9.69±0.93	28	12.0	6	3	0.5	22	2.93	1	20	7	1			1	Not specified	LPxTG	0.883	0.917	Lp_3115
12	Lp_1448	MKKLMCLFGVIGGLVFMSWTSPSVQA-TA	9.26±0.62	26	9.3	3	2	0.7	23	2.52	1	18	7	1			1	None	Membrane	0.673	0.567	Lp_1448
13	Lp_1278	MLKLIKQRLVWGLVLTATVSGVLGCNVAAHA-TS	9.09±0.89	31	9.8	8	3	0.4	23	2.18	1	22	9	1				TSSKADEV	Secretory	0.725	0.708	Lp_1278
14	Lp_3452	MKKGLLVTGRLLMTVIGCLIISNWGWFIATQA-AT	7.91±1.03	32	10.1	10	3	0.3	22	2.76	1	23	7	1				None	N-term	0.634	0.606	Lp_3452
15	Lp_3093	MNFKSQNPIDASQSERFKLYKSGKLWLVAGLTFFSFLGGSVLNDTNVHA-DA	7.73±0.57	49	9.4	24	3	0.1	25	2.02	1	18	25	1				None	N-term	0.39	0.329	Lp_3093
16	Lp_0141	MKKLTSVVATLALALPLAASPFAMTTTANA-AT	7.71±1.32	30	10.0	3	2	0.7	27	2.72	1	23	7	1				TTANAATV	Secretory	0.889	0.888	Lp_0141
17	Lp_3065	MPNKWWRLILGVMLVLSWAIPVRA-AT	7.49±0.65	24	12.0	7	2	0.3	17	3.29	1	18	5	1	1			Not specified	LPxTG	0.74	0.774	Lp_3065
18	Lp_3676	MRRLLTGTLVVGGLLLVVCLMAVNGQA-KV	7.02±0.96	27	10.4	3	2	0.7	24	3.36	1	20	4	1				VNGQAKVV	Secretory	0.726	0.68	Lp_3676
19	Lp_3411	MTKRMSFKFKWVALVATLIVGIGSWQVLAHA-DS	5.68±0.98	31	11.3	10	4	0.4	21	3.07	1	23	7	1				VLAHADSS	N-term	0.865	0.811	Lp_3411
20	Lp_2977	MKKMMRWLGAILVMISGLSAVVPAQA-TN	5.44±1.26	26	11.2	6	3	0.5	20	2.59	1	18	7	1			1	Not specified	LPxTG	0.853	0.798	Lp_2977
21	Lp_2958	MKLSKRGLFWLLGLVSFAILLLFSQPLGAQA-AT	5.23±0.4	31	11.2	6	3	0.5	25	3.08	1	23	7	1	1			Not specified	LPxTG	0.927	0.902	Lp_2958
22	Lp-1097	MKTKLIALIGVIAMVTGMAVFLNQRQQSAKA-TT	4.92±0.91	31	11.3	4	2	0.5	27	3.17	1	18	5	1				QSAKATTT	Secretory	0.582	0.565	Lp-1097
23	Usp45	MKKKIISAILMSTVILSAAAPLSGVYA-DT	4.45±0.12	27	10.0	4	3	0.8	23	2.56	1	23	5	1				SGVYADTN	Secretory	*0.773*	*0.754*	Usp45
24	Lp_3413	MNGWNKKLLLGMLTLIMTLLAGMTVIGHA-AD	4.28±0.19	29	10.0	7	2	0.3	22	2.70	1	20	7	1	1			Not specified	LPxTG	0.783	0.695	Lp_3413
25	Lp_0469	MQTKKWIRGLVCGLALLGGWSLALPVHA-TS	3.98±0.79	28	10.1	8	3	0.4	20	2.54	1	18	7	1				LPVHATSV	Secretory	0.629	0.609	Lp_0469
26	Lp_3178	MQKTKAWYQKWWAWVLILLGIFFVISLVNSPQDGKNSQA-AT	3.42±0.52	39	9.8	10	3	0.3	29	3.39	1	18	12	1				SQAATTKK	Secretory	0.549	0.479	Lp_3178
27	Lp_2578	MRRKLVGYMLSMLTVILALFMLGSTAHA-KE	3.41±0.56	28	11.0	4	3	0.8	24	2.91	1	23	5	1	1			Not specified	LPxTG	0.908	0.834	Lp_2578
28	Lp_2173	MRKLIKACGLMVISMLVGLGIVTSALA-AK	2.99±0.32	27	10.1	6	3	0.5	21	2.57	1	20	7	1				TSALAAKT	Secretory	0.815	0.793	Lp_2173
29	Lp_2174	MGVFRMRWKQIGQWWLLVLALLLGGLLGSLPGQA-AS	2.55±0.25	34	12.0	9	3	0.3	25	3.16	1	20	13	1			1	None	Membrane	0.771	0.713	Lp_2174
30	Lp_2588	MRKKWRWLLLALTGIFFLMFGPPLVSQA-RN	2.55±0.28	28	12.0	6	4	0.7	22	2.58	1	20	7	1	1			Not specified	LPxTG	0.83	0.756	Lp_2588
31	Lp_1303	MFKVGRTWVFTAMASFVLTAAPQLVSHADTVSA-DT	2.19±0.47	33	8.5	6	2	0.3	27	2.00	1	20	7	1			1	None	Membrane	0.836	0.764	Lp_1303a
32	Lp_3064	MRNRRVKWLLILVFGILGLLNLLPLEGNA-AT	2.03±0.11	29	11.7	7	4	0.6	22	3.42	1	19	7	1				LEGNAATL	Secretory	0.844	0.776	Lp_3064
33	Lp_2645	MKIGMTKKVVTSLLLSTALLPMLSGKADTASA-NQ	1.86±0.25	32	10.0	8	3	0.4	24	2.06	-	0	-	-				DTASANQK	Secretory	0.814	0.806	Lp_2645
34	Lp_3116	MKKFVLGLMISAGLLGATTVVANA-DT	1.47±0.19	24	10.0	3	2	0.7	21	2.40	1	18	5	1				VVANADTT	Secretory	0.898	0.814	Lp_3116
35	Lp_1643	MRNRLNRLGLESKSHYKLYKSGRRWVAASITVFSVGIGLTFSQVEQVKA-AT	1.28±0.17	49	11.0	24	8	0.3	25	1.99	1	20	25	1	1			None	Membrane	0.371	0.359	Lp_1643
36	Lp_3072	MMLTKRWWQLGIGLLVGLVSLSWGRTGSA-NT	1.25±0.62	29	12.0	6	2	0.3	23	2.57	1	18	7	1			1	Not specified	LPxTG	0.632	0.551	Lp_3072
37	Lp_3679	MLVRVSKLVSGILVASSLLVSSGVIAQA-DT	1.12±0.4	28	11.0	7	2	0.3	21	2.72	1	23	5	1				VIAQADTP	Secretory	0.903	0.866	Lp_3679
38	Lp_1524	MQTLKPFESIWNFVLLLLVSYPILGAFA-WF	1.11±0.06	28	5.8	5	1	0.2	23	2.70	1	18	10	1				None	Membrane	0.459	0.494	Lp_1524
39	Lp_0353	MKNKYSAKLGLTTLTVIFGLGLTPIANA-KS	1.08±0.27	28	10.0	8	3	0.4	20	2.38	1	20	7	1				PIANAKSI	Secretory	0.721	0.716	Lp_0353
40	Lp_3073	MKKTLLGLLFSAALIATSGLTASA-AD	0.79±0.24	24	10.0	3	2	0.7	21	2.59	1	18	5	1				LTASAADT	N-term	0.944	0.905	Lp_3073
41	Lp_2940	MSKALKIVMGITMLTGGIMAQKMTVHA-AE	0.78±0.04	27	10.3	6	2	0.3	21	2.11	-	0	-	-	1			Not specified	LPxTG	0.579	0.45	Lp_2940
42	Lp_3450	MWKGPKQILVALTALMGLWAGTAQIQA-AD	0.73±0.13	27	10.0	6	2	0.3	21	2.77	-	0	-	1				TAQIQAAD	Secretory	0.779	0.655	Lp_3450
43	Lp_1229	MLKKDNFGEHKTHYKLYKCGKNWAIMGITLVSLGVGTVTMTRAAA-AD	0.66±0.42	45	9.8	21	6	0.3	24	2.31	1	23	21	1	1			Not specified	LPxTG	0.528	0.485	Lp_1229
44	Lp_3067	MKSMLGHLLMAGTLLLAGSTPIVANA-AE	0.63±0.16	26	8.5	2	1	0.5	24	2.40	-	0	-	1				IVANAAEN	Secretory	0.94	0.809	Lp_3067
45	Lp_3678	MRLKVVIQFIRLMGVSLGFSILIGQTGVSA-NR	0.63±0.07	30	12.0	11	3	0.3	19	2.40	1	23	7	1			1	None	Membrane	0.744	0.659	Lp_3678
46	Lp_3453	MKKYLGTLVAGMTLMAGLPLVGQA-AD	0.61±0.06	24	9.7	3	2	0.7	21	2.01	1	18	5	1				None	N-term	0.782	0.629	Lp_3453
47	Lp_3114	MQRRRLQRAQLTEKRTYKMYKKGRLWLIAGLSTFTLGASLLPMTGRA-DT	0.46±0.16	47	11.9	24	9	0.4	23	1.66	1	23	23	1	1			Not specified	LPxTG	0.44	0.379	Lp_3114
48	Lp_1256	MRLKIRGRWLLGLMLGMGLWGPVTVHA-AT	0.25±0.08	27	12.3	8	4	0.5	19	1.98	1	18	7	1				VTVHAATN	Secretory	0.568	0.613	Lp_1256
49	Lp_2978	MKKVVGSLLMTSTILLGALTPIVGHA-AD	0.24±0.04	26	10.0	3	2	0.7	23	2.17	1	20	5	1				VGHAADGE	Secretory	0.871	0.776	Lp_2978
50	Lp_0923	MNKKLLYTSITTAALFVGTQLGVNNAQA-DT	0.21±0.02	28	9.7	4	2	0.5	24	1.90	-	0	-	1	1			Not specified	LPxTG	0.817	0.668	Lp_0923
51	Lp_0197	MRLIDFKTWIMGTAAMLTLIVTNQTVSA-AD	0.16±0.01	28	8.5	7	1	0.1	21	2.27	-	0	-	-	1			Not specified	LPxTG	0.839	0.599	Lp_0197
52	Lp_2795	MEQVKKRYKMYKSGKMWLFAGITLVTLNMNVVTGRA-DE	0.15±0.04	36	10.4	15	5	0.3	21	2.12	1	20	13	1	1			Not specified	LPxTG	0.613	0.623	Lp_2795
53	Lp_3015	MKKLLTTILTTSAATVGLLLAGTVSAHA-DA	0.14±0.02	28	10.0	3	2	0.7	25	2.23	1	23	4	1		1		VSAHADAT	Secretory	0.844	0.809	Lp_3015
54	Lp_0277	MKNTFKKLTLTLMAAGTLFATSVSASA-DT	0.12±0.05	27	10.3	7	3	0.4	20	1.84	-	0	-	-				VSASADTT	Secretory	0.862	0.831	Lp_0277
55	Lp_1449	MFNRFCLSTGLAILGLMATSLTAIG-AT	0.12±0.01	25	8.0	4	1	0.3	21	2.29	1	20	5	1				LTAIGATT	Secretory	0.756	0.719	Lp_1449
56	Lp_3127	MNKRKIITNNPPKWHLITGIAATILASIILTNQDAFA-AT	0.11±0.04	37	10.3	15	5	0.3	22	2.58	1	18	13	1	1			Not specified	LPxTG	0.839	0.77	Lp_3127
57	Lp_1447	MAKFRRLVLLSLSLGLALAGGCRSPDALA-AT	0.11±0.03	29	10.8	6	3	0.5	23	2.36	-	0	-	-	1			Not specified	LPxTG	0.772	0.719	Lp_1447
58	Lp_2796	MRLIVRSVRLFLKKWGITINYRESEVKCYKMYKSGKMWLLASASLLLLNTQLLTAHA-DE	0.1±0.01	57	10.3	36	8	0.2	21	2.33	1	18	38	1	1			Not specified	LPxTG	0.317	0.29	Lp_2796
59	Lp_3412	MMVLLQVIAAGATVSLGADMTAQA-AT	0.09±0.02	24	3.8	0	0		24	2.51	-	0	-	-				TLPQLTFA	Secretory	0.526	0.59	Lp_3412
60	Lp_3043	MRFPKRIFSTLATISLTTSFGLLSANA-ST	0.08±0.03	27	12.0	6	3	0.5	21	1.60	1	19	7	1				ANASTKAT	Secretory	0.866	0.816	Lp_3043
61	Lp_2976	MMRWLLIRGLLGLLIGLGPSIGGA-AQ	0.08±0.01	24	12.0	8	2	0.3	16	2.48	-	0	-	1	1			Not specified	LPxTG	0.814	0.67	Lp_2976
62	Lp_3414	MTKSRTLKAAMLVSVASIACLSFNNLGASA-DT	0.08±0	30	10.0	8	3	0.4	22	2.36	-	0	-	1				LGASADTL	Secretory	0.903	0.88	Lp_3414
63	Lp_2925	MYKIGRRWAFACAVILTMGTTTLVARA-DD	0.07±0.01	27	10.9	7	3	0.4	20	2.51	1	19	7	1	1			Not specified	LPxTG	0.829	0.599	Lp_2925
64	Lp_1450	MKKTVLTATMLLSLLGMPAVMATA-DS	0.07±0	24	10.0	3	2	0.7	21	1.91	-	0	-	-				VMATADST	Secretory	0.946	0.871	Lp_1450
65	Lp_0869	MTSMAYRTPPFISPFAIVSIVLALGATNVVVNKTTHTVNG-AK	0.05±0.02	40	10.0	7	1	0.1	33	3.09	1	23	10	1				KTTHTVNG	Secretory	0.562	0.629	Lp_0869
66	Lp_0800	MNRFITSKQHYKMYKKGRFWVFAGITVATFTLNPLISRA-DT	0.05±0	39	11.2	18	7	0.4	21	2.03	1	18	19	1	1			Not specified	LPxTG	0.668	0.568	Lp_0800
67	Lp_3014	MKKLVSTIVTTSAAAAGLLFAGVLNANA-DS	0.04±0.01	28	10.0	3	2	0.7	25	2.36	1	23	4	1		1		None	N-term	0.923	0.832	Lp_3014
68	Lp_2845	MKIKNALLTSLAATGLLALGTTVANA-DT	0.04±0.01	26	10.0	4	2	0.5	22	2.17	1	18	7	1		1		TVANADTV	Secretory	0.878	0.859	Lp_2845
69	Lp_2175	MRTFKLMTGLSGGATLLSLALMPLAALA-AG	0.03±0.02	28	11.0	5	2	0.4	23	2.32	1	20	7	1				LAAGPGTA	Secretory	0.839	0.788	Lp_2175
70	Lp_3001	MIKPRVLTTLLVCSAILTTTVTPAVA-AV	0.03±0.02	26	9.5	5	2	0.4	21	2.54	-	0	-	-	1			Not specified	LPxTG	0.908	0.885	Lp_3001
71	Lp_2486	MTGDSVYRVKMYKDGKRWVYAGATTLALAAGLVFANVNASA-DT	0.03±0.01	41	9.7	17	3	0.2	24	2.60	1	23	18	1	1			Not specified	LPxTG	0.611	0.55	Lp_2486
72	Lp_1124	MKRNSQQSTTVDHYKMFKDGKHWVYAGITIAGLGSTLMLTTNALA-AT	0.03±0.01	45	9.7	22	5	0.2	23	1.61	1	23	22	1	1			Not specified	LPxTG	0.465	0.393	Lp_1124
	pNuc-cyt		0.02±0.01																			
73	Lp_0302	MKIKNLVLSSTAALALFAISTTVANA-DT	0.02±0.02	26	10.0	4	2	0.5	22	2.38	1	20	5	1		1		TVANADTY	Secretory	0.894	0.88	Lp_0302
74	Lp_3059	MRYTRGKWRVTNPKVWLFSSVLILGWRIVPTVAQA-SE	0.02	35	12.0	14	5	0.4	21	2.32	-	0	-	-	1			Not specified	LPxTG	0.837	0.826	Lp_3059
75	Lp_2162	MSQAHTTGKVLASTVGAVGLLLATGQAANA-AS	0.01±0.01	30	8.5	9	2	0.2	21	2.51	-	0	-	1		1		QAANAASI	N-term	0.758	0.738	Lp_2162
76	Lp_3421	MKYIRDILFGVIIIKVKVNIKQVMLGTAALAGMLVTSQAVANA-DS	0.01±0.01	43	10.2	21	4	0.2	22	2.77	-	0	-	1		1		AVANADSM	N-term	0.524	0.507	Lp_3421
77	Lp_0304	MKIKNALLSTAAATASLFAIGATAQA-AT	0.01±0	26	10.0	4	2	0.5	22	1.87	1	18	7	1		1		ATAQAATV	Secretory	0.888	0.903	Lp_0304
78	Lp_2847	MKIKHLLLSSLATTGAIALGATAAKA-DT	0.01±0	26	10.3	5	3	0.6	21	1.80	1	18	7	1		1		TAAKADTV	Secretory	0.853	0.793	Lp_2847
		Mean values		31	10.2	8.3	3	0.4	23	2.52										0.739	0.689	
		Totals:									62			69	25	8	8					

[a] The N-domain is defined as the N-terminal part of the SP, ending at the last positively charged residue. Histidine was defined as positive.
[b]The maximum hydrophobicity of the SPs was estimated using the ProtScale program (Gasteiger et al, 2005) at the ExPASy Server (http://www.expasy.ch/tools/protscale.html). The windows size was set to 7 residues (default setup).
[c]Motifs found in the Secretome database entry for L. plantarum WCFS1 (see www.cmbi.ru.nl/secretome and Ref. 18). Proteins for which no anchoring motif is indicated are predicted to be secreted. Lp_1524 is not included in the Secretome database, meaning that the only localization prediction for this protein comes from LocateP.

[d] Signal Peptide cleavage site and subcellular localization prediction found in the LocateP database (http://www.cmbi.ru.nl/locatep-db/cgi-bin/locatepdb.py); see Zhou et al., 2008 (Ref. 54) for details. LocateP does not provide predicted N-terminal cleavage sites for proteins that are anchored via the sortase pathway and the LPXTG motif (hence "not specified"). "N-term" indicates proteins that possibly are N-terminally anchored through their (non-cleaved) signal peptides. "Membrane" indicates proteins annotated by LocateP as "Multi-transmembrane". Note that LocateP predictions are fully automatic and expected to be less accurate than the predictions in the Secretome database; see text for details.
