# Supplementary material for: Genome-wide analysis of signal peptide functionality in Lactobacillus plantarum WCFS1
Source: BMC Genomics. 2009 Sep 10;10:425. doi: 10.1186/1471-2164-10-425 (PMC2748100; doi:10.1186/1471-2164-10-425)
Supplement: Additional file 2 — WebLogos of signal peptides. Frequency plots made with WebLogo [34], based on multiple alignment of the sequence starting13 residues upstream of the predicated cleavage site and ending two residues downstream. [file 1471-2164-10-425-S2.pdf]

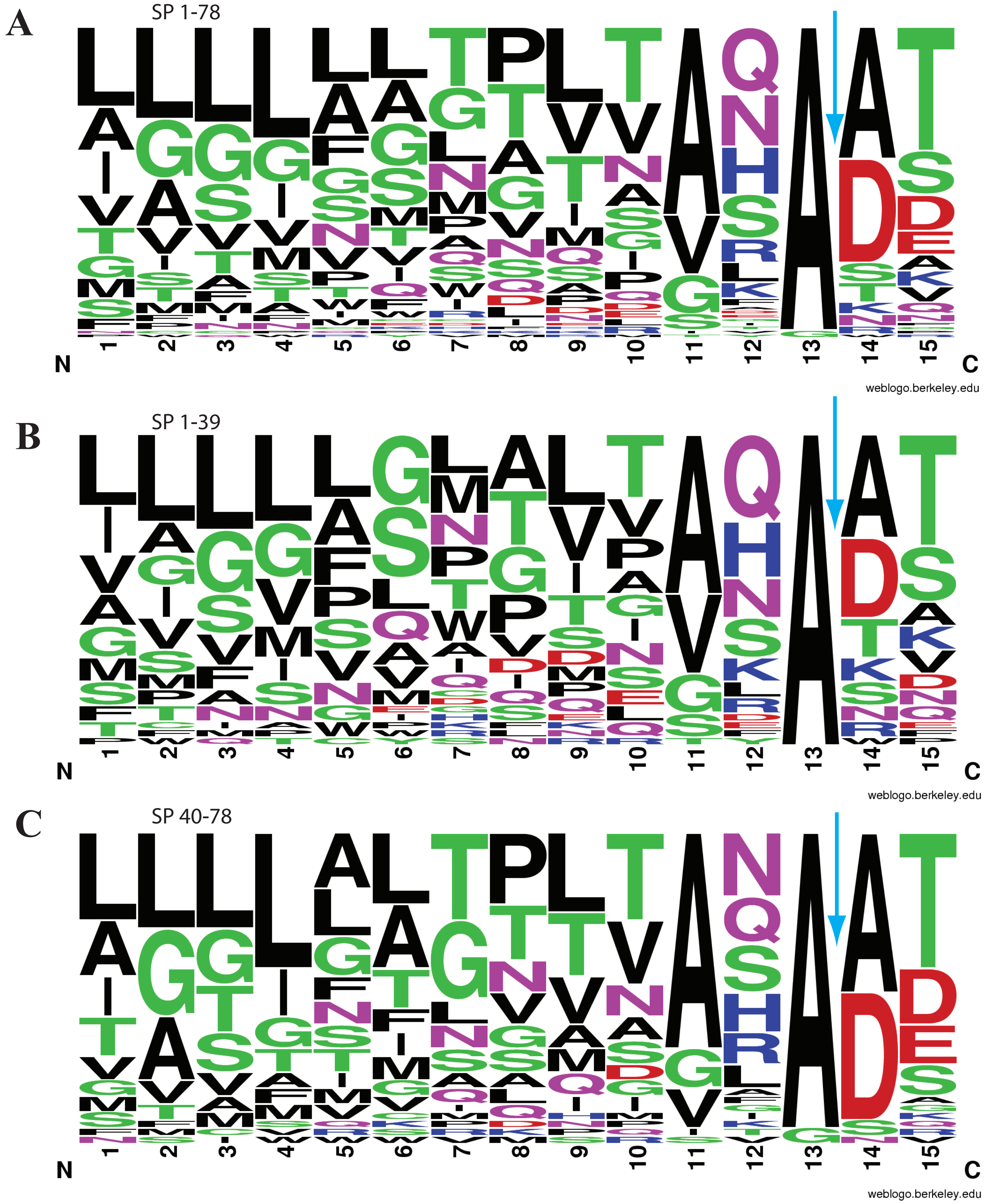
A
SP 1-78
N
C
weblogo.berkeley.edu
B
SP 1-39
N
C
weblogo.berkeley.edu
C
SP 40-78
N
C
weblogo.berkeley.edu

D

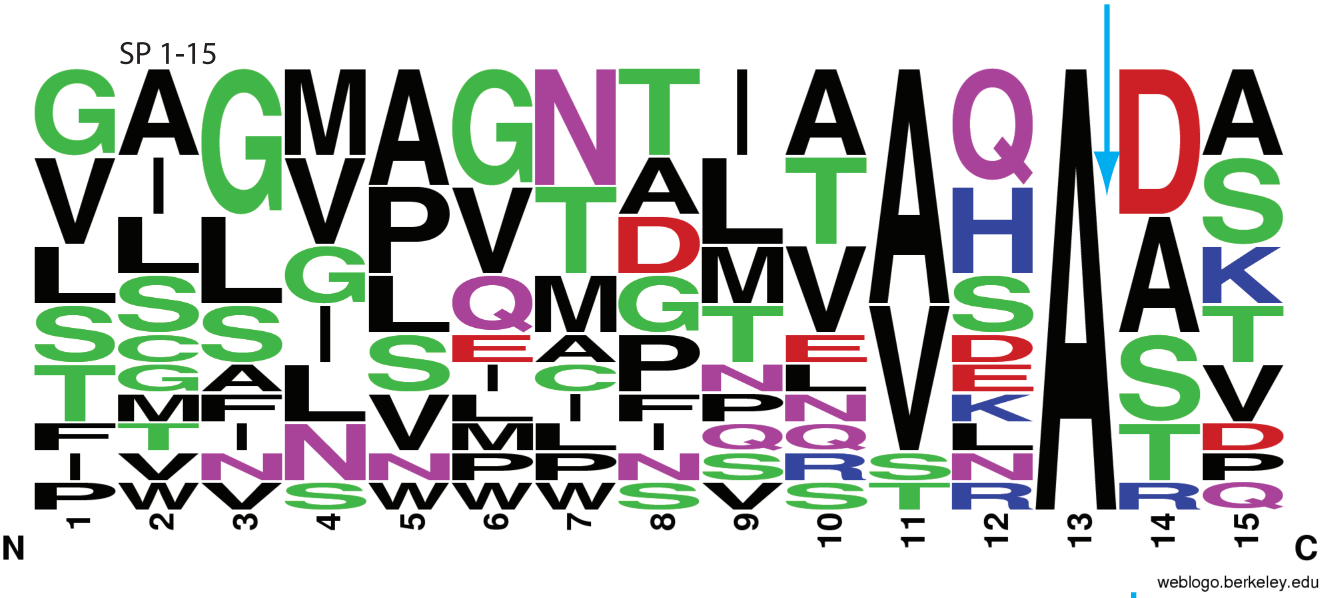

E

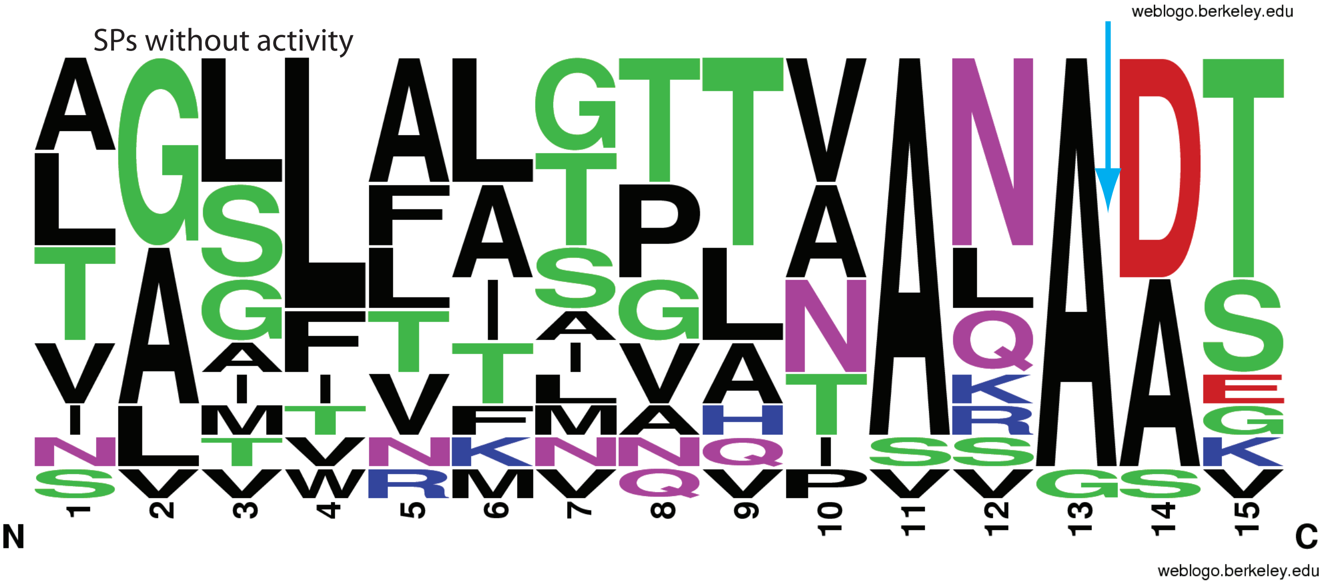

Figur S1. Frequency plot using WebLogo (Crooks et al., 2004), based on multiple alignment of 13 residues upstream of the predicated cleavage site and two residues downstream. Subgrouping of SPs is based on their performance with the NucA reporter protein. A, composition map of all SPs in the study; B, composition map of the 39 best performing SPs; C, the 39 least performing SPs; D, the 15 best performing SPs; E, the 14 SPs that resulted in no significant extracellular NucA activity. The blue arrow indicates the predicated cleavage site.
