# Supplementary material for: Genome-wide analysis of signal peptide functionality in Lactobacillus plantarum WCFS1
Source: BMC Genomics. 2009 Sep 10;10:425. doi: 10.1186/1471-2164-10-425 (PMC2748100; doi:10.1186/1471-2164-10-425)
Supplement: Additional file 3 — SDS-PAGE analysis of L. plantarum containing various constructs for amylase secretion. Coomassie stained and Silver-stained SDS-PAGE gels of cell extracts and cell free supernatants of L. plantarum WCFS1 harbouring various amylase constructs. [file 1471-2164-10-425-S3.pdf]

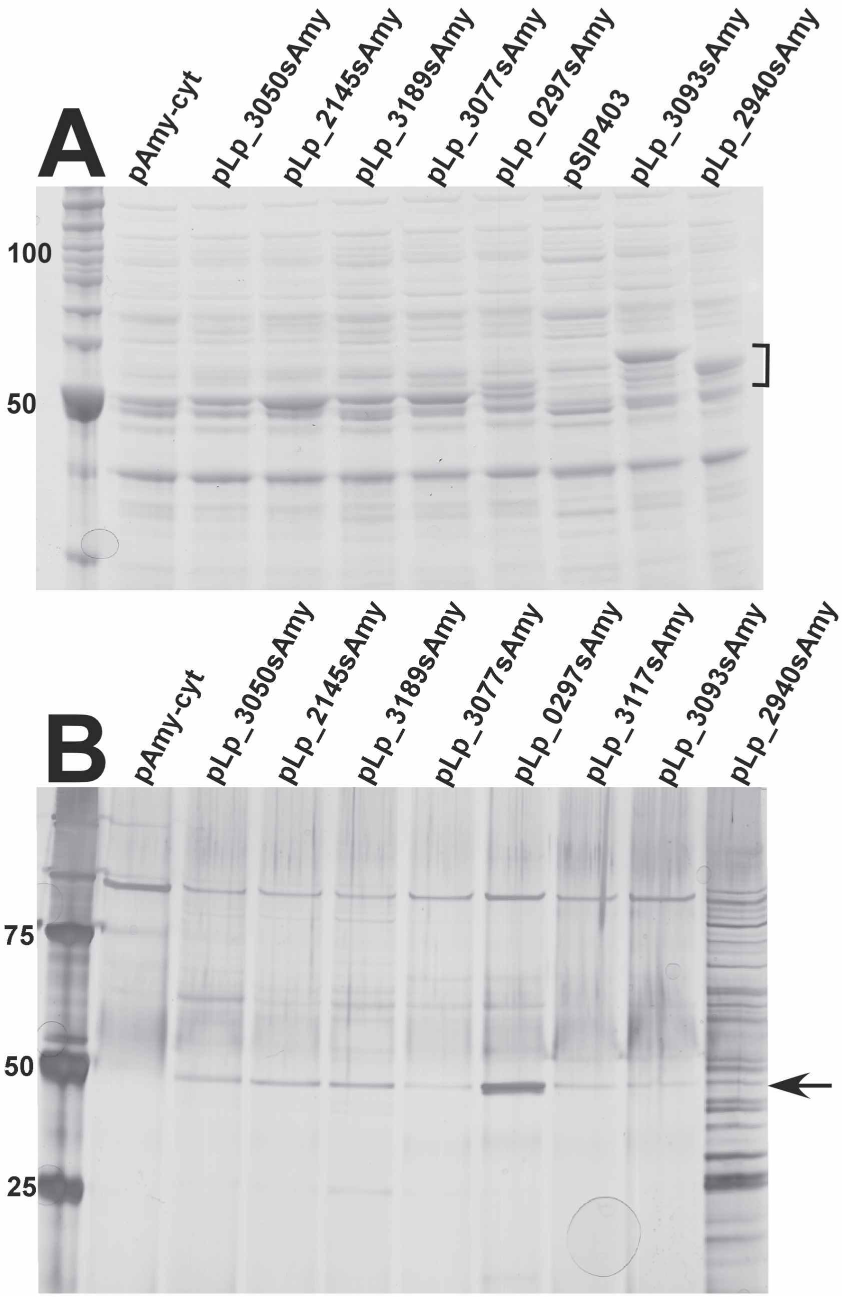

Figure S2. Coomassie stained (A) and Silver-stained (B) SDS-PAGE gels of cell extracts (A) and cell free supernatants (B) of L. plantarum WCFS1 harbouring various amylase constructs. Lanes are marked with the names of the plasmid constructs; pSIP403 is a construct lacking the amyA gene (Sørvig et al, 2003); pAmy-cyt is a construct driving cytoplasmic production of AmyA. The molecular masses of marker proteins are given in kDa. The bracket (panel A) and the arrow (panel B) indicate amylase bands. Note that the position of the amylase precursor in the gel of panel A is partly hidden by background bands (i.e. bands also seen in the lane marked pSIP403). The position of the precursor band varies because of differences in SP length and, apparently, because the different precursors behave slightly differently in terms of unfolding during sample preparation. The supernatant fraction of L. plantarum harbouring pLp_2940sAmy shows a protein pattern similar to what is observed for cell extracts (i.e. intracellular proteins), indicating cell lysis. All harvested supernatant fractions for this construct (three replicates) showed the same pattern.
