# Supplementary material for: Genome-wide analysis of signal peptide functionality in Lactobacillus plantarum WCFS1
Source: BMC Genomics. 2009 Sep 10;10:425. doi: 10.1186/1471-2164-10-425 (PMC2748100; doi:10.1186/1471-2164-10-425)
Supplement: Additional file 4 — List of primers. Primers used in real-time PCR experiments and for PCR-amplification of signal peptides from genomic DNA of L. plantarum WCFS1. [file 1471-2164-10-425-S4.pdf]

Table S2. Primers used in RT-PCR experiments and for PCR-amplification of signal peptides from genomic DNA of *L. plantarum* WCFS1. All primers are named after gene name. F or R denote forward or reverse primers, respectively. All forward primers have inserted an *Nde*I site (CATATG) and reverse primers a *Sal*I site (GTCGAC).

Name	Primer sequence (5'→3')
0141F	CCATATGAAAAAATTAACATCAGTCGTTGCAAC
0141R	CGTCGACCGTGGCGGCATT
0197F	CCATATGCGATTAATCGATTTCAAAACTTGGAT
0197R	GGTCGACGTCCGCCGCACT
0277F	GCCATATGAAAAACACATTCAAAAAACTAACCCTTAC
0277R	GGTCGACCGTGTCAGCAGAGGC
0297F	CCATATGAAAGAAGTAAGGTTTTGGGGACT
0297R	GGTCGACTACGTCAGCCTTACTAAC
0353F	CATATGAAGAATAAGTACAGTGCTAAGTTAGGT
0353R	GTCGACCGATTTTGCGTTGGCAAT
0373F	CATATGTATACGGAAAACACGGGGAAAC
0373R	GTCGACTGATGAAGCATCAACCAC
0469F	CATATGCAGACGAAGAAGTGGATTC
0469R	GTCGACACTAGTTGCGTGTACT
0600F	CATATGATGAAACATAAAAATTTATTATTATTAATTATAATGAGC
0600R	GTCGACCTTATCCGCTTCAACTC
0800F	CATATGAATA GATTTATAAC GAGCAAGCAG CATT
0800R	GTCGACCGTATCTGCCCGACTT
0869F	CATATGACATCGATGGCTTATCGAACAC
0869R	GTCGACCTTGGCGCCATTGAC
0923F	CATATGAATAAGAAATTATTGTATACCAGTATTACCACT
0923R	GTCGACCGTGTCGGCCTGCGC
0946F	CATATGAAACCAAACAATGTTAATAATCAGAATAAACGTC
0946R	GTCGACGTCGGCAGCCGCC
1097F	CATATGAAAACGAAATTAATTGCACTGATTGGAGT
1097R	GTCGACGGTCGTCGCTTTGGC
1124F	CATATGAAAAGGAATTCACAACAATCAACCAC
1124R	GTCGACCGTTGCTGCTAAGGC
1229F	CATATGTTAA AAAAGGATAA TTTTGGAGAG CATAAAACAC
1229R	GTCGACATCTGCTGCGGCGGC
1256F	CATATGAGATTGAAAATAAGGGGACGCTGGCTGTT
1256R	GTCGACCGTCGCCGCATGCAC
2977F	CATATGAAGAAAATGATGCGGTGGTT
2977R	GTCGACATTGGTTGCCTGTGCT
3001F	CATATGATAAAGCCGCGTGTTTTAACGAC
3001R	GTCGACTACTGCAGCGACGGC
3014F	CATATGAAAAAACTTGTAAGTACAATCGTAACTACCT
3014R	GTCGACTGAGTCGGCGTTAGCATT
3015F	CATATGAAAAAATTATTAACCACAATCTTAACAACATCC
3015R	GTCGACTGCGTCGGCGTGTGC
3043F	CATATGCGCTTTCCAAAAAGAATCTTTTCAACAC
3043R	GTCGACTGTTGATGCGTTGGCACT
3050F	CATATGAAAAAATTTAACTTTAAAACCATGTTGCTATTAGT
3050R	GTCGACCTTGGAGGCCTGGGC
3059F	CATATGCGAT ATACACGGGG CAAAT
3059R	GTCGACTTCACTTGCTTGAGCCAC
3064F	CATATGCGTAACAGACGAGTAAAATGGTTAC
3064R	GTCGACAGTCGCGGCGTTAC

3065F	CCCATATGCCGAATAAATGGTGGCGATT
3066R	GTCGACTGTTGCCGCCCGCACT
3067F	GCCATATGAAGTCAATGTTAGGACACTTATT
3067R	GTCGACTTCTGCAGCATTGGCAAC
3072F	CGCATATGATGTTAACAAAGAGATGGT
3072R	GTCGACTGTGTTAGCCGAACCCGTT
3073F	GCCATATGAAAAAAACATTATTAGGACTTTTATTCTC
3073R	GTCGACATCGGCCGCACTC
3077F	GCCATATGAAGAAATTGATGATTGCTGTAACT
3077R	GTCGACAGTCGCCGCATTCGCT
3093F	GCCATATGAATTTTAAATCACAAAATCCAATTGATGC
3093R	GTGCACGCTGATGCTGTCGAC
3114F	CATATGCAAAGACGACGCTTACAACGAGC
3114R	GTCGACCGTGTCTGCCCGCC
3115F	GCCATATGCAGAAACGATTGCGATTATC
3115R	GTCGACCTGATCAGCAGAGGCT
3116F	GCCATATGAAGAAATTCGTTTTGGGATTAATGAT
3116R	GTCGACCGTATCAGCATTAGCC
3117F	GCCATATGCGGCGAATATGTAAAGTATTAATGGTAATT
3117R	GTCGACCGGCGCGGCCAGC
3127F	GCCATATGAACAAGCGAAAAATTATCACTAATAACC
3127R	GTCGACAGTGGCCGCAAAGGC
3189F	GCCATATGAGATTTGCGGGAAAGTTAAAGAAAGTT
3189R	GTCGACACTGGCTGCTTGGGC
3411F	GCCATATGACGAAACGAATGAGTTTCAAATTTAAAT
3411R	GTCGACACTGTCAGCGTGCGC
3412F	GCCATATGATGGTATTGTTACAAGTCATAGC
3412R	GTCGACCGTAGCTGCTTGAGC
1303aF	CATATGTTTAAAGTCGGCCGTACTTGGGTATTT
1303aR	GTCGACAGTATCCGCCGAAACC
1447F	GCCATATGGCTAAATTCAGGCGTCTAGT
1447R	GTCGACGGTAGCCGCTAGGGC
1448F	GCCATATGAAAAAATTGATGTGCTTATTCGGCGTAATT
1448R	GTCGACAGCGGTGGCCTGGAC
1449F	GCCATATGTTTAACCGCTTTTGTCTTAGCACG
1449R	GTCGACCGTTGCCCCGATAGC
1524F	GCCATATGCAAACACTTAAGCCCTTCGAAAGT
1524R	GTCGACAAACCAGGCAAACGCAC
1643F	GCCATATGCGGAATCGTTTAAATAGGTTGGGAT
1643R	GTCGACCGTTGCGGCTTTCACTT
2145F	GCCATATGAAAAAAATTAATAAGTTGATGATCTTAGGCATGC
2145R	GTCGACGGCGCTAGCATGC
2162F	CATATGTCACAAGCACATACAACGGGC
2162R	GTCGACTGAGGCAGCATTGGCAGCT
2173F	CATATGAGAAAGTTGATCAAGGCTTGTGGCCTAAT
2173R	GTCGACTTTGGCTGCTAAGGCACT
2174F	GCCATATGGGGGTGTTTAGAATGCGAT
2174R	GTCGACAGATGCCGCTTGACC
2175F	GCCATATGCGAACTTTTAAATTAATGACCGGGTT
2175R	GTCGACTCCGGCCGCTAAC
2486F	CATATGACCGGTGATTCGGTTTATCGGGT
2486R	GTCGACAGTATCAGCAGAAGCATTAACGTT
2645F	GCCATATGAAAATTGGAATGACAAAAAAAGTAGTAAC
2645R	GTCGACTTGATTTGCACTTGCCGT

2795F	CATATGGAACAAGTGAAGAAACGGTACAAGAT
2795R	GTCGACTTCATCTGCTCGTCC
2796F	GCCATATGAGATTAATCGTTAGGTCAGTT
2796R	GTCGACTTCATCCGCATGTGC
2845F	GCCATATGAAAATTAAAAATGCCCTATTAACTTC
2845R	GTCGACGGTATCGGCATTAGC
2847F	GCCATATGAAAATTAAACACCTATTATTATCCTCAT
2847R	GTCGACCGTATCGGCCTTAGC
2925F	GCCATATGTACAAAATTGGCCGTCGCT
2925R	GTCGACATCATCAGCCCGAGC
2940F	GCCATATGTCAAAAGCGCTTAAGATAGT
2940R	GTCGACTTCGGCTGCATGTAC
2976F	GCCATATGATGCGATGGCTGTTGATAC
2976R	GTCGACCTGAGCAGCTCCC
3413F	GCCATATGAACGGCTGGAATAAGAAGC
3413R	GTCGACGTCAGCAGCATGGC
3414F	GCCATATGACAAAGTCACGTACGCTC
3414R	GTCGACGGTGTCAGCGGAAGC
3421F	GCCATATGAAGTACATACGTGACATTC
3421R	GTCGACTGAATCGGCATTGGC
3450F	GCCATATGTGGAAGGGACCGAAACAAAT
3450R	GTCGACGTCAGCAGCCTGAAT
3451F	GCCATATGAAAAAAATATTTTGGCAGTTAATTGCC
3451R	GTCGACGTCATCCGCTCTAGC
3452F	CATATGAAAAAGGGATTGTTGGTAACAGGT
3452R	GTCGACCGTTGCAGCTTGC
3453F	CATATGAAAAAATATCTCGGAACTCTCGT
3453R	GTCGACATCTGCAGCTTGGC
3676F	CATATGAGAAGGTTACTTACTGGCAC
3676R	GTCGACAACCTTTGCCTGCCCAT
3678F	GCCATATGAGATTGAAAGTGGTTATCCAATTC
3678R	GTCGACCCGATTAGCCGACAC
3679F	CATATGTTGGTAAGAGTTAGTAAGTTAGTCAGT
3679R	GTCGACCGTATCTGCCTGAGC
2578F	CATATGCGAA GAAAGCTAGT TGGGT
2578R	GTCGACTTCTTTAGCATGTGCAG
1278F	CATATGTTAAAGTTAATCAAACAACGGTTGGTATGGGGA
1278S	GTCGACACTGGTCGCGTGTGC
3178F	CATATGCAAAAAACGAAAGCTTGGT
3178S	GTCGACTGCTTGTGAGTTTTTACC
2958F	GGGGCATATGAAGTTAAGTAAGCGGGGACT
2958R	GTCGACAGTCGCGGCTTGGGC
2588F	CATATGCGCAAAAAAATGGCGATGGT
2588R	GTCGACGTTGCGGGCCTGAC
0304F	CATATGAAGATTAAGAATGCTTTATTATCAACTGCTGCCG
0304R	GTCGACCGTAGCAGCTTGAGCGGT
1450F	CATATGAAAAAAACAGTGCTTACCGC
1450R	GTCGACTGAATCGGCGGTTGC
0302F	CATATGAAGATCAAAAACCTTGTATTATCAT
0302R	GTCGACAGTATCAGCGTTGGC
2978F	CATATGAAAAAAGTAGTAGGCAGTTTACTCATGACC
2978R	GTCGACATCGGCCGCGTGAC
GyrAF	CCCGACAGCAACGTCTTCA
GyrAR	GGCAGCTGGCGTTTGTTT

NucAF	GGTCAACCAATGACATTCAGACT
NucAR	ACTTGCTTCAGGACCATATTTCTC
